# DNA Tetrominoes: The Construction of DNA Nanostructures Using Self-Organised Heterogeneous Deoxyribonucleic Acids Shapes

**DOI:** 10.1371/journal.pone.0134520

**Published:** 2015-08-10

**Authors:** Hui San Ong, Mohd Syafiq Rahim, Mohd Firdaus-Raih, Effirul Ikhwan Ramlan

**Affiliations:** 1 Natural Computing Laboratory, Department of Artificial Intelligence, Faculty of Computer Science and Information Technology, University of Malaya, 50603, Kuala Lumpur, Malaysia; 2 School of Biosciences and Biotechnology, Faculty of Science and Technology and Institute of Systems Biology, Universiti Kebangsaan Malaysia, 43600, Bangi, Malaysia; Imperial College London, UNITED KINGDOM

## Abstract

The unique programmability of nucleic acids offers alternative in constructing excitable and functional nanostructures. This work introduces an autonomous protocol to construct DNA Tetris shapes (L-Shape, B-Shape, T-Shape and I-Shape) using modular DNA blocks. The protocol exploits the rich number of sequence combinations available from the nucleic acid alphabets, thus allowing for diversity to be applied in designing various DNA nanostructures. Instead of a deterministic set of sequences corresponding to a particular design, the protocol promotes a large pool of DNA shapes that can assemble to conform to any desired structures. By utilising evolutionary programming in the design stage, DNA blocks are subjected to processes such as sequence insertion, deletion and base shifting in order to enrich the diversity of the resulting shapes based on a set of cascading filters. The optimisation algorithm allows mutation to be exerted indefinitely on the candidate sequences until these sequences complied with all the four fitness criteria. Generated candidates from the protocol are in agreement with the filter cascades and thermodynamic simulation. Further validation using gel electrophoresis indicated the formation of the designed shapes. Thus, supporting the plausibility of constructing DNA nanostructures in a more hierarchical, modular, and interchangeable manner.

## Introduction

Deoxyribonucleic acid (DNA) is an interesting molecule to be exploited as programmable substrates at the nanometre scale [1–7]. DNA nanostructures can be constructed by utilising the canonical interactions that define how the base components in the nucleotide chains interact via Watson-Crick base pairing, to form double stranded DNA molecules [8]. Fundamentally, base pairings are essential in providing a minimal programmability in constructing sophisticated molecular nanostructures with various structural designs [6, 7, 9–14]. Due to their ability to interact with other functional molecules, synthetic nanostructures have been designed for various applications such as biosensors [15], for drug delivery [16, 17], as bio-imaging probes [18, 19] and as substrates for bio-sensing and bioassays [20–23].

Most of the current DNA nanostructures reported were constructed using various approaches, including the DNA origami technique [13, 24, 25] and the use of DNA sticky ends to connect different molecular tiles [26]. Although successful in providing proof-of-concepts for designing programmable DNA blocks, these conventional approaches were restricted because they required carefully designed and well-defined structures to ensure preordained error-less organisation. These structures can only be generated through a set of distinct and restrictive sequences. Therefore, any base mutations or mispairings would result in incompatible binding and thus, non-conformity of the desired structures.

In resolving this issue, we investigated the plausibility of utilising the self-organising property of DNA molecules to oversee the formation of DNA nanostructures. This minimises the complexity of designing DNA strands and the self-assembly (folding) errors usually encountered during the formation of such structures. This is achieved by allowing competition between various heterogeneous shapes to occur without any pre-specified binding instructions (i.e., orchestration of blocks [27] instead of total programmability). While homogenous blocks (identical and symmetrical DNA shapes) as fundamental units ensure conformity in the structure formation [28, 29], our heterogeneous blocks are unique in being able to promote self-organisation and thus better optimisation of structure formation. The assembly using heterogeneous blocks allows the formation of structures to rely purely on natural processes without any interference from predefined sets of instructions. This increases the flexibility of constructing DNA nanostructures as the formation of the structures are achieved through any combination of competing DNA shapes. These DNA shapes are designed with stable free energy to keep the rigidity of the later formed structures intact. The minimum or maximum number of DNA shapes involved in each formation can be optimised during the design stage. The comparison between the conventional approaches (DNA Origami and SST) with the proposed method is presented in Table 1.

**Table 1:** Comparison between DNA Origami, Single stranded DNA Tiles (SST) and DNA Tetrominoes.

	DNA Origami	Single stranded DNA Tiles (SST)	DNA Tetrominoes
**Schema**	Each distinct structure [13, 20, 25, 30] requires a new scaffold routing design and the synthesis of different sets of staple strands.	Modular assembly: Every single component in the structure can be included, excluded or replaced without changing the remaining structure [31]. Homogeneous: Components that make up the nanostructures are always uniform (standardized components).	Modular assembly: Every single component in the structure can be included, excluded or replaced without changing the remaining structure. Heterogeneous: Components used to make up a larger structure need not always be the same (A larger structure can be made using various Tetris shapes)
**Degree of Difficulty in Sequence Design**	Restrictive: Meticulous sequences design (each staple strand is targeted to a specific location in the scaffold).	Moderate: Design of sequences for uniform motif is straightforward, however individualised sequences design is required to enable intermolecular binding between domains in every SST motif [28, 29, 31].	Easy: Each shape is independent of the overall structure design (and only conform to its individual shape), then the sequence generation is context free. Matching dangling ends are randomised to each shape afterwards.
**Programmability**	Hard: DNA Origami uses a long scaffold as construction material. Therefore, in order to build a gigadalton nanostructure, it would requires a scaffold (over 1 megabase) which is mechanically fragile and difficult to synthesize [32]	Moderate: One standardized length with four domains is used throughout structure. (e.g. 42 nucleotides single stranded DNA was used to build a molecular tube [28], 32 nucleotides single stranded DNA were used to build 3-D shapes [31]	Loose: Any Tetris shape can combine together with different combinations to generate larger structures. The users only need to program the sticky ends of each Tetris shape to be compatible.

The fabrication of DNA nanostructures starts with the sequence design phase. This is commonly conducted using computational tools [33–36] to generate sequences with minimum free energy (MFE) and minimisation of sequence symmetry [33, 37]. Programs such as Tiamat [33] and SEQUIN [38] were developed to prohibit sequence symmetry from occurring throughout the DNA construct (i.e., to avoid undesired pairing within the design sequences). On the contrary, our autonomous protocol allows the structure to have sequence symmetry at tolerable degrees. It allows the structures to form, subjected to the occurrences of some unwanted aggregates. This is necessary to handle the formation of structures under undesirable and uncontrollable physicochemical conditions. This would be applicable for a specific scenario where structures are built *in-situ* inside living cells, as compared to the conventional method of building the structures externally (thus requiring a complicated delivery mechanism afterwards) [39]. One such scenarios could be where we have DNA nanorobots with acceptable levels of stability inside a cellular environment, however with the drawback of time limitation to resist enzymatic degradation [40, 41].

**Fig 1:**
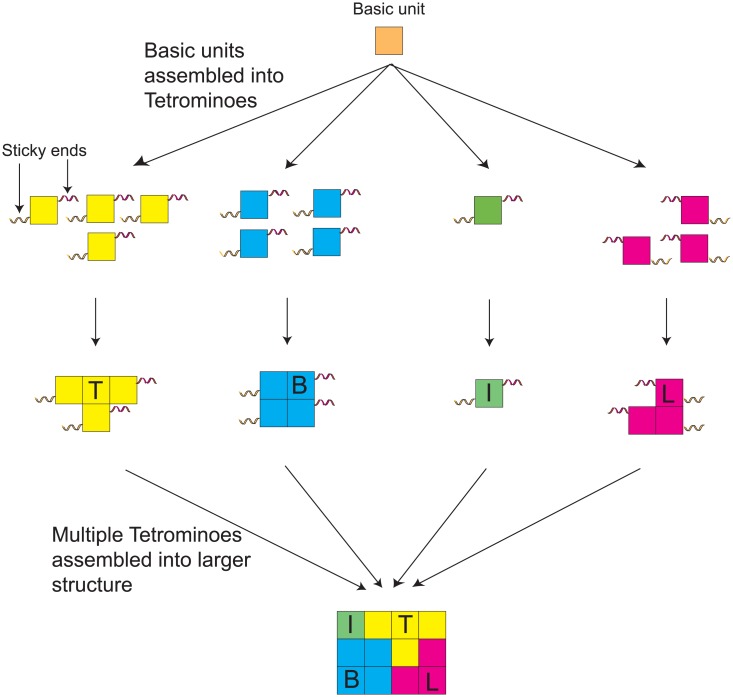
Conceptual illustration of the hierarchical schematic to form supra molecular structures using DNA Tetrominoes.

In this study, we propose a new hierarchical schema of assembling supra-molecular structures. As a basis, we use two single stranded DNAs to form our elementary blocks. Then, using these elementary blocks, we constructed four distinct DNA shapes (called DNA Tetrominoes; as the shapes resemble some basic shapes available in the game Tetris). These shapes then would further assemble into the intended supra molecular structures (as illustrated in Fig 1).

Compared to conventional DNA origami approaches, during the final assembly phase, in our proposed schema, the formation of the supra structures would entirely be dependable on the DNA shapes themselves. This is possible because in fabricating a particular DNA structure, our autonomous protocol will generates an *N* amount of DNA shapes with *M* amount of sequences for each shape (*N* and *M* are dynamic variables that can be customised according to the user). Through *in-silico* optimisation, the most preferred shape and sequence combinations will take precedence, however, since the shape and sequence combinations are modular, each shape and sequence are interchangeable without affecting the desired structures. The schema promotes a complete outlook of the shapes and sequences landscape necessary in designing any DNA structures. The proposed schema also relief the constraints of identifying and specifying base pairing dependencies mandatory in constructing any DNA structures, therefore allowing for a more flexible DNA structures construction.

## Materials and Methods

An autonomous protocol was developed that comprised of (i) a sequence design pipeline to generate sequences for each DNA Tetris shape and (ii) an optimisation algorithm to mutate sequences that violated the fitness criteria. The DNA Sequence Generator program [42] was embedded inside the sequence design pipeline to facilitate the computational speed of generating the desired sequences. The optimisation algorithm was built entirely using Tool Command Language (TCL) [43–45] and Perl version 5.12.4 and the complete protocol was tested under the Unix environment in Mac OS X, version 10.7.5.

### Protocol (i): Single Stranded Deoxyribonucleic Acid (ssDNA) Sequence Design

The DNA Sequence Generator (DSG) available from the CANADA package [42, 46] was used to generate the initial sequences of single stranded deoxyribonucleic acids (ssDNA). This program uses a fully automatic graph based approach to create uniqueness within a pool of sequences. The default parameters were applied (as suggested in [42, 46]), with the exception of sequence length, which was set to 25 nucleotides.

#### Modification of ssDNA to Block Structures

**Fig 2:**
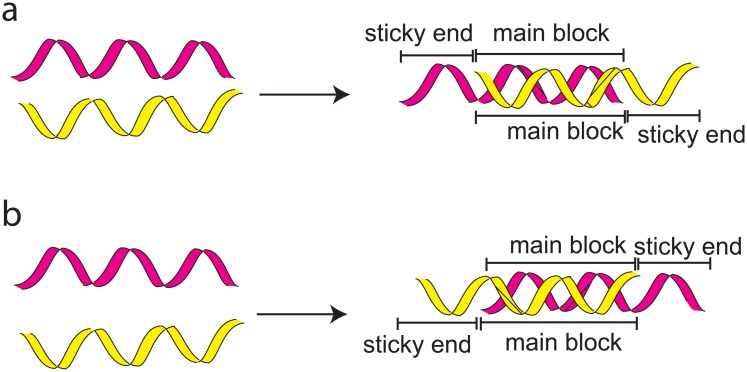
Basic blocks used in designing the structures a) Type-1 and b) Type-2. The total length of each ssDNA is 25 nucleotides; whereby each strand is compartmentalised into a main block (15 nucleotides) and two sticky ends (10 nucleotides).

The ssDNAs generated from the DSG were then modified into a block structure of double stranded DNA (dsDNA). The main block and sticky ends are 15 and 10 nucleotides respectively. There are two basic types of blocks (type-1 and type-2) as depicted. Every two ssDNAs were treated as a block. For each pair, sequences in the main block were modified to complement with each other to form block structures as shown in Fig 2.

#### Stacking and Merging of Blocks

**Fig 3:**
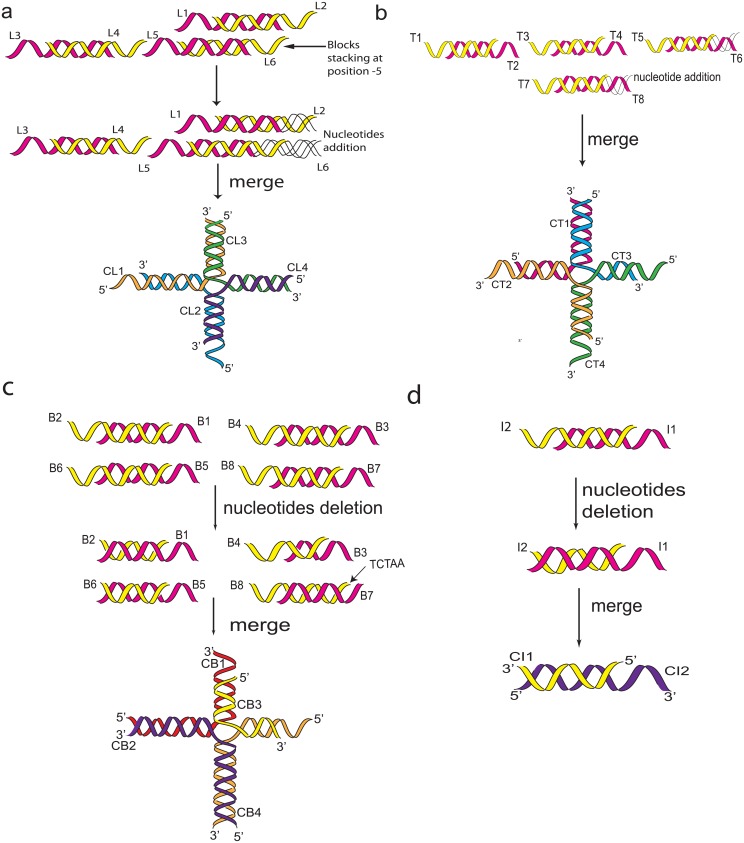
(3a) Schematic illustration for L-shape formation using 3 blocks or 6 DNA strands (L1-L6). These strands were then subjected for modification (insertion of 10 and 15 nucleotides to L1 and L5, insertion of 5 nucleotides to L6) and block stacking (Block L5-L6 was stacked on position -5 (to the left) relative to Block L1-L2). After modification, these 3 blocks then merged to form 4 long strands (CL1-CL4). (3b) Schematic illustration for T-shape formation using 4 blocks or 8 DNA strands (T1-T8). These strands were then subjected for nucleotides modification (insertion of 10 and 20 nucleotides to form blunt end on T6 and sticky ends on T8. After modification, it was then merged to form 4 newly combined strands (CT1-CT4). (3c) Schematic illustration for B-shape formation using 4 blocks or 8 DNA strands (B1-B8). These strands were then subjected for modification such as deletion (Deletion of 10 nucleotides on strand B2-B4, B6 and B7) and fragment shifting (fragment “TCTAA” shift from strand B7 to B8). Thereafter, blocks were merged to form into 4 long strands (CB1-CB4). (3d) Schematic illustration for I-shape formation using a single block or 2 DNA strands (I1, I2). These strands were subjected for modification, deletion of 10 nucleotides occurred on I2 sticky ends. Following modification, CI1 and CI2 are the new strands.

A single crossover between blocks was implemented into the design by stacking one DNA block upon another block. DNA strands were subjected for modification such as the position of block stacking, nucleotide shifting, sequence insertion and deletion were incorporated into the optimisation algorithm to ensure a greater versatility in nucleotide combinations for the resulting structures. Each basic Tetris shape (with an exception of the I-Shape) was built from six ssDNAs or eight ssDNAs, which were then merged to form four long continuous ssDNAs. Meanwhile, the I-Shape was formed using two ssDNAs (Fig 3).

### Protocol (ii): Optimisation of DNA sequences

Sequences generated from the pipeline were optimised further to increase the feasibility of the desired Tetris structures formation in the laboratory. The optimisation algorithm, which incorporated four fitness criteria (Table 2), was used to calculate the penalty scores for all the generated sequences in each population.

**Table 2:** Fitness evaluation criteria for sequence optimisation implemented in Protocol (ii).

Fitness criterion	Description
Base pairing at false binding sites	The detection program exhaustively looks for maximum consecutive base pairing at unwanted positions (also known as False Binding Site or FBS), FBS_max_ = 6.
Thermodynamics free energy	The calculation of thermodynamics incorporated the use of program *AllSub* and *DuplexFold* [47] Thermodynamics free energy of intra-molecular pairing must be higher compared to inter-molecular pairing.
Percentage of Guanine-Cytosine (GC) content	Percentage of GC must be in the range of 40% to 70% inclusive.
No existence of G4 pattern	No existence of GGGG pattern was allowed in the sequence.

The penalty score increases (i.e., increment by a point) whenever the sequence does not pass any of the evaluation criteria (otherwise, the penalty score will be nil). If the total penalty score of the four fitness criteria exceeds 0, the sequence will undergo a mutation process. The algorithm will randomly select new nucleotides to replace the existing nucleotides (at any random position) in the mutation permissible region. Only a single nucleotide will be mutated at a time; the penalty score will be recalculated and mutations will be conducted repeatedly until the penalty score becomes nil.

#### Base Pairing at False Binding Sites (FBS)

As a general rule during DNA assembly, it is crucial for DNA sequences to form base pairings exactly at the pre-defined positions. At the same time avoiding pairings at unwanted positions (mispairing). This is also known as "binding specificity". Unfortunately, such false-binding sites (FBS) could not be completely avoided; otherwise the sequence diversity would be extremely low. As a consequence, base pairings at false-binding sites were limited to shorter lengths so that the thermodynamic stability [48–50] of the false-binding sites is predicted to have low energy and accordingly, low probability of hybridisation. Therefore, this criterion was included as a crucial filter and was intended to detect the longest complementary region that existed between two sequences. In this work, base pairing at a false-binding site is defined as the occurrence of two sequences that form base pairings at unwanted positions.

The detection program was written using Perl version 5.12.4 and was processed using the following three scripts: (i) *FindStartPosition*.*pl*, (ii) *CleanEmptyPosition*.*pl* and (iii) *GetLongestComplement*.*pl*. The calculations were conducted by aligning a query sequence against the remaining corresponding target sequences. The query was shifted a nucleotide at a time towards the 3’ terminal to search for any complementary nucleotides in the target. During each shift, if a nucleotide from a target strand is complementary with the nucleotide from the query strand; the False Binding Sites (FBS) score increases by one, (if and only if the longest complementarity at unwanted position is more than six, otherwise the FBS-score remained unchanged). The final FBS-score represents the longest consecutive stretch of complementary bases that was detected between the two strands.

The first script (*FindStartPosition*.*pl*) was employed to find all positions that have a minimum of seven consecutive complementary (Q_min_) nucleotides between the query and target sequence. It listed out every start position that matches to the minimum complementary bases. The output of *FindStartPosition*.*pl* listed every start position in the query ($QStart) and target ($TStart). The function of *CleanEmptyPosition*.*pl* is to remove the query, which does not meet the threshold value of at least seven consecutive matching nucleotides. The *GetLongestComplement*.*pl* script was then executed to obtain the longest matching complementary sequence using the start position output from the first script. Parameters are dynamic can be customised to any design specification (e.g. minimum consecutive complementary might be different depending on design of the DNA shapes).

#### Thermodynamic Energy of inter-molecular and intra-molecular DNA pairings

The thermodynamic energy for a DNA sequence to form self-folding (intra-molecular) and double stranded folding (intermolecular) were calculated using the "*AllSub*" and "*DuplexFold*" programs available in the RNAstructure package [47]. The program “*AllSub*” is selected to generate all possible low free energy structures of a given DNA sequence. The program “*DuplexFold*” is used to predict the lowest free energy structure for two interacting sequences with a constraint of not allowing any intra-molecular base pairs to occur. Default parameters were selected with the exception of the RNA/DNA option, which was set to only DNA.

This fitness evaluation required the free energy of “*AllSub*” to be higher (less negative) than the energy of “*DuplexFold*” (more negative). This was to ensure a relatively more stable structure when bindings occurred between two ssDNAs as compared to the stability of ssDNA self-folding. This is to ensure that correct base-pair formation for inter-molecular assembly occurs.

#### G4 Pattern

The sequence design was prevented from having a G4 sub-sequence pattern because such sequences are favourable to form an unintended four-stranded G4 DNA structure [51].

#### Percentage of GC Content

The number of Gs and/or Cs of oligonucleotides is between 40% and 70% inclusive. The GC content was calculated by obtaining the number of GC versus the total nucleotide content.

#### Mutation

Mutations were exerted on DNA strands if the total score from all four fitness criteria are more than zero. The regions for the mutations to be exercised were based on 2 conditions depending whether a forbidden region exists (Condition 1 if the region exists and condition 2 otherwise). Variable *$MutateRegion* is a list of nucleotide positions that allow mutations to occur, while variable *$ForbidPosition* is a list of nucleotide positions that does not allow mutations to occur mainly because these nucleotides are hybridised with the previous strands. The formula for determining the mutation regions is *$MutateRegion = $AllPosition—$ForbidPosition*. For instance, the calculation of the *$MutateRegion* if there is forbidden region is depicted in Table 3. In this instance, sequence CB2 has 30 nucleotides, and the nucleotides numbered 16–30 from CB2 are complementary with nucleotides numbered 1–15 from strand CB1.

**Table 3:** Condition 1 (the existence of the forbidden region).

*$AllPosition*	1 2 3 4 5 6 7 8 9 10 11 12 13 14 15 16 17 18 19 20 21 22 23 24 25 26 27 28 29 30
*$ForbidPosition*	16 17 18 19 20 21 22 23 24 25 26 27 28 29 30
*$MutateRegion*	1 2 3 4 5 6 7 8 9 10 11 12 13 14 15

Table 4 depicts an example of the mutating region (*$MutateRegion*) where the forbidden region is non-existence. In this instance, sequences in CL1 do not have complementary binding with any sequences. The length of the molecule is 35 nucleotides.

**Table 4:** Condition 2 (the non-existence of the forbidden region).

*$AllPosition*	1 2 3 4 5 6 7 8 9 10 11 12 13 14 15 16 17 18 19 20 21 22 23 24 25 26 27 28 29 30 31 32 33 34 35
*$MutateRegion*	1 2 3 4 5 6 7 8 9 10 11 12 13 14 15 16 17 18 19 20 21 22 23 24 25 26 27 28 29 30 31 32 33 34 35

Therefore, in order for a mutation to occur, a position will be randomly selected, identified as X in *$MutateRegion* and X will be replaced with a randomly selected nucleotide, N_New_.

### Protocol for Laboratory Validation of the Constructed DNA Tetris Blocks

#### DNA Annealing

Oligonucleotides were purchased from Integrated DNA Technologies Pte Ltd. Complexes of shapes were formed by mixing stoichiometric quantities of each strand at 0.5 μM concentration in a buffer consisting of 40 mM Tris base, 2.5 mM EDTA, and 13 mM MgCl_2_. Then, the complexes were formed by annealing the reaction mixture for three hours from 90°C to 4°C in an Eppendorf Mastercycler Pro S thermocycler (Eppendorf, Hamburg, Germany). To form individual shapes, 4 oligonucleotides were mixed stoichiometrically in a buffer containing 40 mM Tris base, 2.5 mM EDTA, and 13 mM MgCl_2_. The final concentration of oligonucleotides was set to 0.5 μM. The solution containing DNA sequences were not treated with any DNA polymerases, to ensure that they were held together only by non-covalent interactions (e.g. hydrogen bonds and base stacking).

#### Gel Electrophoresis

The results of annealing reactions were analysed by electrophoresis using 12% non-denaturing 0.75 mm thick polyacrylamide gel (29:1 acrylamide: bisacrylamide). The running buffers contained 1X TBE (89 mM Tris base, 89 mM Boric acid and 2 mM EDTA pH8.3) and 10 mM MgCl_2_. The loading buffers contained 30% glycerol and 0.25% Bromophenol blue tracking dye. The gels were run at approximately 12 V/cm^-1^ for 4 hours (for L-, B-, T-, I-Shapes) at 4°C and then stained with the GelRed Nucleic Acid gel stain (Biotium, US).

## Results and Discussion

**Fig 4:**
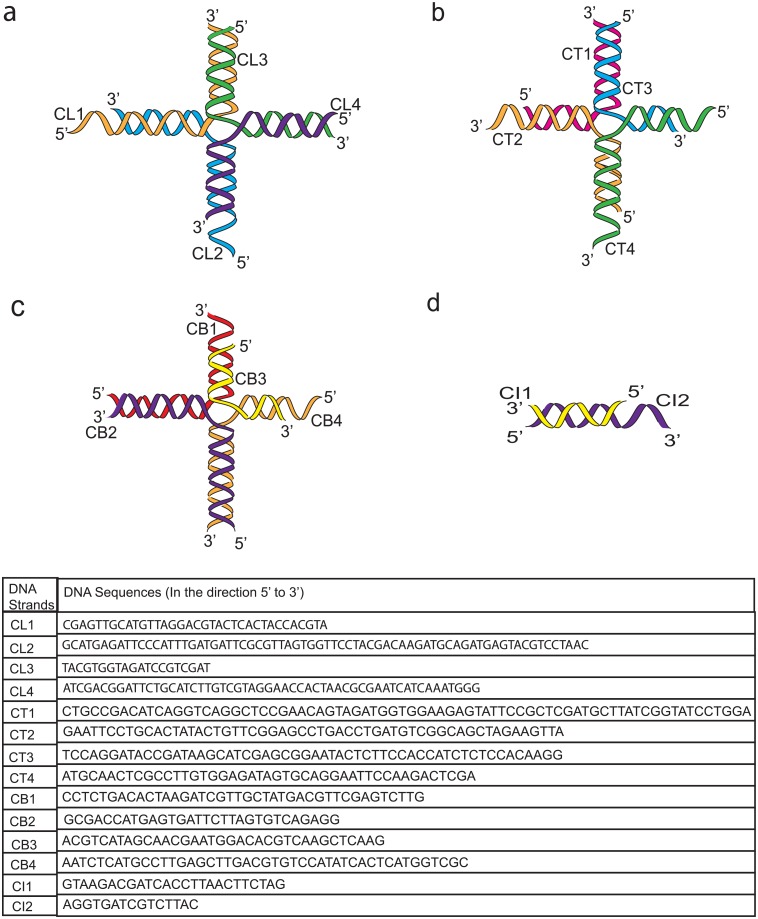
Conceptual illustrations of the DNA sequence in forming a) L-shape, b) T-shape, c) B-shape and d) I-shape. L-Shape, T-Shape and B-Shape are made up of 4 single stranded DNA oligomers (CL1-CL4, CT1-CT4, CB1-CB4). I-Shape is made up of 2 single stranded DNA oligomers (CI1, CI2).

Four Tetris shapes; L-Shape, T-Shape, B-Shape and I-Shape were successfully generated. The B-Shape, and T-Shape were built from four blocks; the L-Shape from three blocks while the I-Shape used only a single block. Two types of blocks were introduced, the Type-1 was used to build the L- Shape, while the Type-2 was used to build the T-Shape, B-Shape and I-Shape. To ensure that molecular optimisation can be approximated accordingly, merged blocks were further subjected to insertion, deletion and the shifting of nucleotides between strands. Two neighbouring blocks were linked using the existing sticky ends while a single crossover was utilised to ensure the linkage between two blocks formed when the two blocks are stacked on top of each other. The merging of short sequences from 3 blocks (L-Shape) and 4 blocks (T-Shape, B-Shape) resulted in four long stretches of DNA sequences (Fig 4).

### Analysis of Laboratory Validation

**Fig 5:**
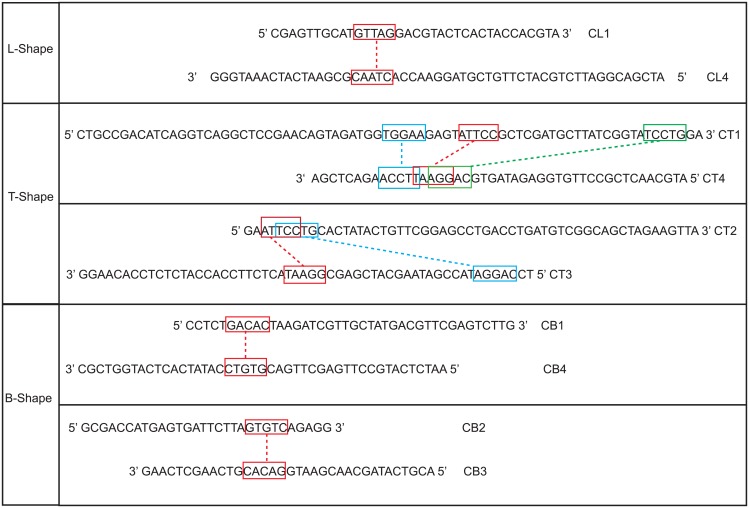
List of mispairing bases (i.e., binding between bases with incorrect base positions).

In this work, our autonomous pipeline generates 500 populations for each individual shape. A random sample from each shape was taken for gel electrophoresis study to detect the assembly of the ssDNA components into the Tetris structures. Previous study reported that five nucleotides [11] are sufficient to create the possibility of binding, although six [52] or more are more commonly used; anything less than five is regarded as insufficient to form stable binding. Using the sequences from the random sample, in Fig 5 we highlighted mispairings of bases that might influence the result of our laboratory validation.

**Fig 6:**
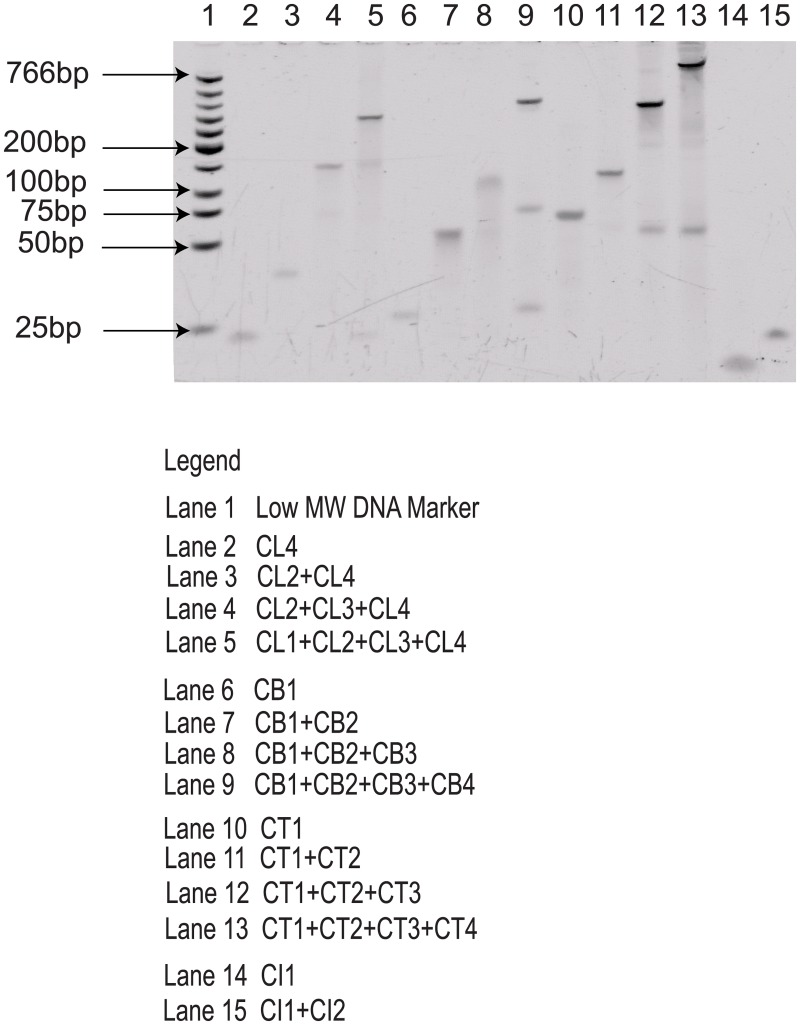
Gel electrophoresis showed the band increment for the sequence used to form the Tetris shape. Gel electrophoresis was conducted on 12% non-denaturing PAGE gel.

To fully implement the proposed hierarchical schematic, a less stringent approach was adopted during the sequence design. We allowed mispairing of bases (i.e., complementary binding at incorrect position) to occur in the designed sequences (with subtle limitations) to ensure that the correct bindings would still occur. By referring to the Fig 5 and the resulting gel electrophoresis experiment in Fig 6, we could observed that there are two extra bands appeared below the major bands which are the unwanted aggregates proceeding from the mispairing between CB1-CB4 and CB2-CB3. As for the T-shape, there is an extra band with the same band size observed in both Lane 12 and Lane 13. Similarly, these are the unwanted aggregates derived from the mispairing of CT2-CT3. The complementary binding at the correct position is set at least 10 nucleotides to provide sufficient strength in the structure formation. Supported by the gel electrophoresis results, the formation of the designed DNA Tetris shape is satisfactory except for some minor unwanted aggregates (which is expected due to the allowance of the protocol).

### Analysis of the autonomous protocol

**Fig 7:**
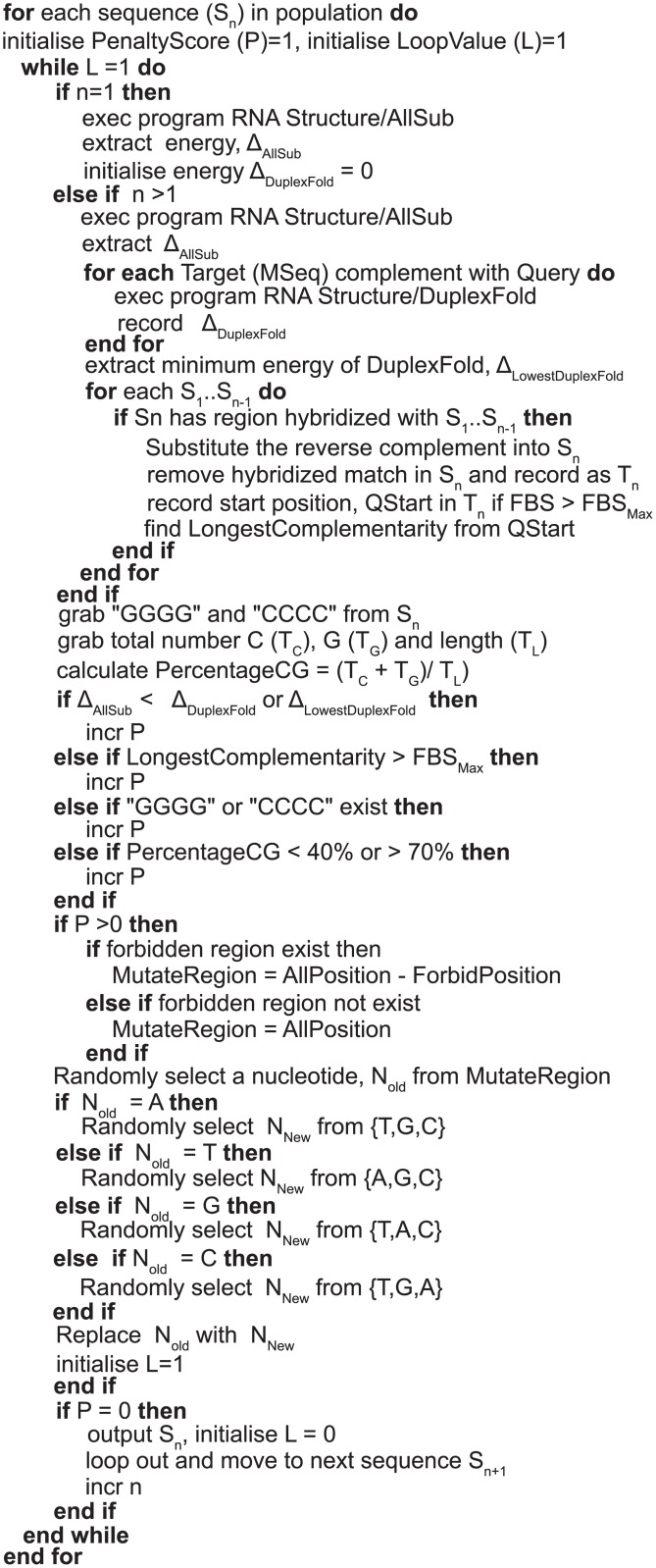
The algorithm for sequence optimisation.

The autonomous protocol optimises the following four parameters (i) FBS_max_ = 6 [52], (ii) thermodynamic free energy = Δ_DuplexFold_ < Δ_AllSub_, (iii) G4 pattern = 0 and (iv) percentage of GC between 40% to 70% inclusive. For each generation, if a sequence does not comply with all four fitness criteria, it will mutate to produce a new sequence and will be re-evaluated. The whole cycle was repeated until the four criteria were satisfied. There are two parts in this algorithm (Fig 7) of fitness evaluation, i.e., the sequence evaluation based on the four parameters and the sequence mutation. The program required two inputs files: (i) Sequence.txt, (A file containing sequences produced from the pipeline to further undergo optimisation) and (ii) DefineSeq.txt (A file that lists all positions of nucleotides that form complementary binding between different DNA strands, listed in Table 5).

**Table 5:** Input file for sequence optimisation, to describe nucleotide positions that form complementarity.

DNA Strands, Curr	CurrLength	CurrStart	CurrEnd	MSeq^a^	MStart^b^	MEnd^b^
CL1	35	NIL	NIL	NIL	NIL	NIL
CL2	70	56	70	CL1	11	25
CL3	20	1	10	CL1	26	35
CL4	55	11	55	CL2	11	55
CL4	55	1	10	CL3	11	20
CT1	75	NIL	NIL	NIL	NIL	NIL
CT2	55	16	45	CT1	1	30
CT3	55	1	45	CT1	31	75
CT4	45	21	35	CT2	1	15
CT4	45	11	20	CT3	46	55
CB1	40	NIL	NIL	NIL	NIL	NIL
CB2	30	16	30	CB1	1	15
CB3	35	1	15	CB1	16	30
CB4	45	31	45	CB2	1	15
CB4	45	11	30	CB3	16	35
CI1	25	NIL	NIL	NIL	NIL	NIL
CI2	15	1	15	CI1	1	15

^a^MSeq is the DNA strand that forms complementarity with Curr

^b^Region between MStart and Mend in MSeq form complementarity with region between CStart and CEnd in Curr. NIL indicates an empty value.

#### Thermodynamics Distribution for the Populations

**Fig 8:**
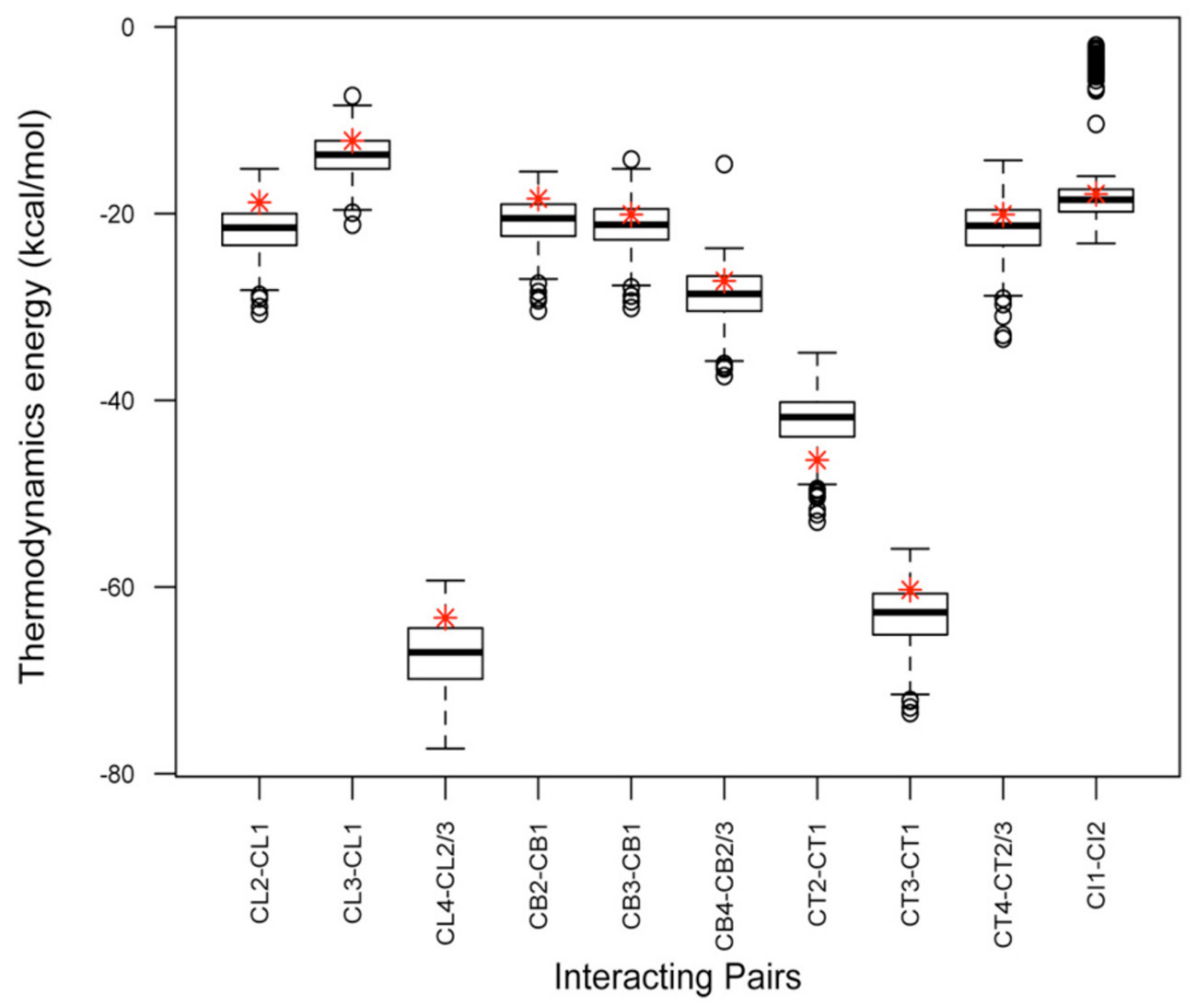
Boxplot showed thermodynamics free energy for 500 populations in each shape. CL4-CL2/3 implies that CL4 hybridised with CL2 and CL3. Therefore, free energy between CL4-CL2 and CL4-CL3 were generated and the lowest energy was used to plot the graph. Red asterisk (*) represent the thermodynamics energy of the strands used for gel electrophoresis study (CL2-CL1: -18.8kcal/mol, CL3-CL1: -12.2kcal/mol, CL4-CL2/3: -63.3kcal/mol, CB2-CB1: -18.4kcal/mol, CB3-CB1: -20.1kcal/mol, CB4-CB2/3: -27.2kcal/mol, CT2-CT1: -46.4kcal.mol, CT3-CT1: -60.3kcal/mol, CT4-CT2/3: -20.1kcal.mol and CI1-CI2: -17.9kcal/mol). Thermodynamics energy were obtained using program “*DuplexFold*” and graphs were generated using R software version 2.15.1 [53]

The thermodynamics free energy for the interaction pairs, Δ_DuplexFold_ was plotted (Fig 8). The distribution of the median (thick horizontal line) showed a relatively uniform distribution between the first and third quartile except for CI1-CI2. This implied that the majority of the populations have relatively similar thermodynamic energy approximations. DNA strands used for gel electrophoresis study (except for pair CT1-CT2) have a higher energy (less negative) than the median of the populations. The red asterisks show the thermodynamics energy for sequences that were selected thorough random sampling for gel electrophoresis study.

#### Number of Iterations

The average number of iterations for B-Shape is 9.9±0.46 cycle, L-Shape 8.5±0.53 cycle, T-Shape 22.4±1.13 cycle and I-Shape 3.1±0.15 cycle. The number of iterations increased linearly as the number of nucleotides in mutated regions increases. This is linear with the number of positions that are permitted to mutate. Furthermore, the number of iterations is also dependent on the complexity of the fitness criteria. However, the approach is still effective and does not require complicated heuristics in order to generate candidate sequences for each DNA Tetris shape. The number of iterations required for each shape is relatively small and the computational process is relatively fast.

Each sequence is defined to be dependent or partially dependent on the nucleotide pattern from the previous sequence using a top-down method (e.g. L1→L2→L3→L4). The optimisation process will only proceed when sequence L1 has satisfied all the four criteria, and then continues with the following sequence (L2) until the designs for all sequences are completed. The lack of positions for sequence mutations such as for the I-shape (made up of two strands) caused the resulting structure to be less susceptible to changes. This is because the sequence arrangement in CI2 depends entirely on CI1 (CI2 not having sticky ends that can be mutated).

## Conclusions

The problem of constructing any DNA nanostructures has always been associated with strict structural and sequence restrictions to ensure that conformity between sequence and its structural formation. This requires extensive knowledge of the molecule. In this work, we propose a simpler hierarchical schema of conducting the design phase. We design DNA shapes (in the form similar to DNA Tetris) that can be assembled into various DNA nanostructures. Our autonomous protocol is constructed in a manner where the parameters employed are flexible for any alterations and has the potential to be extended for complex DNA shape designs. This from-the-ground-up approach allows users with any level of knowledge on DNA molecule to design DNA shapes for the assembly of larger nanostructures due to its modularity. The proposed schema has the potential to become a platform of constructing a more autonomous, self-organised molecular constructs for advanced molecular information processing tasks.
